# Reduced Production of Bacterial Membrane Vesicles Predicts Mortality in ST45/USA600 Methicillin-Resistant *Staphylococcus aureus* Bacteremia

**DOI:** 10.3390/antibiotics9010002

**Published:** 2019-12-18

**Authors:** Somrita Dey, Smitha Gudipati, Christopher Giuliano, Marcus J. Zervos, Jonathan M. Monk, Richard Szubin, Sarah C. J. Jorgensen, George Sakoulas, Andrew D. Berti

**Affiliations:** 1 Department of Pharmacy Practice, Wayne State University College of Pharmacy and Health Sciences, Detroit, MI 48201, USA; somritadey@wayne.edu (S.D.); ek2397@wayne.edu (C.G.); 2 Henry Ford Hospital, Wayne State University School of Medicine, Detroit, MI 48201, USA; sgudipa2@hfhs.org (S.G.); mzervos1@hfhs.org (M.J.Z.); 3 Department of Bioengineering, University of California at San Diego, La Jolla, CA 92093, USA; jonathan.m.monk@gmail.com (J.M.M.); rszubin@eng.ucsd.edu (R.S.); 4 Department of Pharmacy, Mount Sinai Hospital, University Health Network, Toronto, ON M5G 1X5, Canada; sarah.jorgensen@sinaihealthsystem.ca; 5 Division of Host-Microbe Systems & Therapeutics, Department of Pediatrics, University of California at San Diego School of Medicine, La Jolla, CA 92093, USA; gsakoulas@ucsd.edu; 6 Sharp Memorial Hospital, San Diego, CA 92093, USA; 7 Department of Biochemistry, Microbiology and Immunology, Wayne State University College of Medicine, Detroit, MI 48201, USA

**Keywords:** MRSA, membrane vesicles, clinical outcome, biomarker, USA600

## Abstract

Immune biomarkers can stratify mortality risk in staphylococcal bacteremia. Microbial biomarkers may provide more consistent signals during early infection. We demonstrate that in ST45/USA600 bacteremia, bacterial membrane vesicle production in vitro predicts clinical mortality (773 vs. 116 RFU, survivors vs. decedents, *p* < 0.0001). Using a threshold of 301 relative fluorescence units (RFU), the sensitivity and specificity of the membrane vesicles to predict mortality are 78% and 90%, respectively. This platform is facile, scalable and can be integrated into clinical microbiology lab workflows.

## 1. Introduction

*Staphylococcus aureus* is the most common invasive human pathogen and remains a major contributor to infection-related morbidity and mortality [1]. Early identification of patients at high risk of mortality and intensive intervention in this cohort shows promise in reducing overall mortality [2]. However, prognostic biomarkers such as IL-10 and TNFα are produced after the manifestation of symptoms and are not routinely orderable tests in standard clinical microbiology laboratories. The identification of a prognostic microbial factor may provide an earlier tool to recognize patients in need of more intensive anti-staphylococcal therapy. Furthermore, it may provide a more stable value that does not vary based on the patient’s clinical status or time of sampling.

Both the high production of specific pro-inflammatory cytokines and the low production of anti-inflammatory cytokines have been associated with survival during staphylococcal bacteremia [3,4,5]. Most staphylococcal toxins are recognized as potent immunomodulatory effectors [6]. Therefore, counterintuitively, toxin production may contribute to immune recognition, microbial clearance and ultimately favorable clinical outcomes in endovascular infections [7]. Different lineages of clinical *S. aureus* produce distinct immunomodulatory toxins and significant variations in local epidemiology limits the associations between individual effectors and overall survival [8]. Therefore, the ability to predict clinical outcomes based on the production of specific microbial toxin effectors is limited by genetic diversity and functional redundancy.

We hypothesize that membrane vesicles (MV)—detached portions of the staphylococcal membrane enriched with multiple different immunomodulatory effectors [9]—may represent a microbial factor that can be detected early in infection and predict clinical outcomes. These extracellular particles have only recently been recognized in Gram-positive bacteria and perform fundamental roles in mediating staphylococcal–host interactions [10]. Their demonstrated ability to selectively modulate the human immune system has led to their development as a potential platform for anti-staphylococcal vaccine design [11]. We have observed significant heterogeneity in the quantity of MV produced by clinical isolates, even between genetically similar strains. At present, the clinical impact of differential, strain-specific MV production is not known.

Recognizing the presence of endovascular staphylococci and the appropriate immune activation are critical to resolution of staphylococcal bloodstream infection in the host. In the present study, we establish that clinical isolates from survivors of endovascular ST45/USA600 staphylococcal infection produce significantly more MV than isolates from decedents. As such, MV may represent a prognostic microbial biomarker, useful in identifying patients in need of more intensive intervention. To our knowledge, this is the first report to associate the production of MV in vitro with clinical outcomes during endovascular staphylococcal infection.

## 2. Results

**Figure 1:**
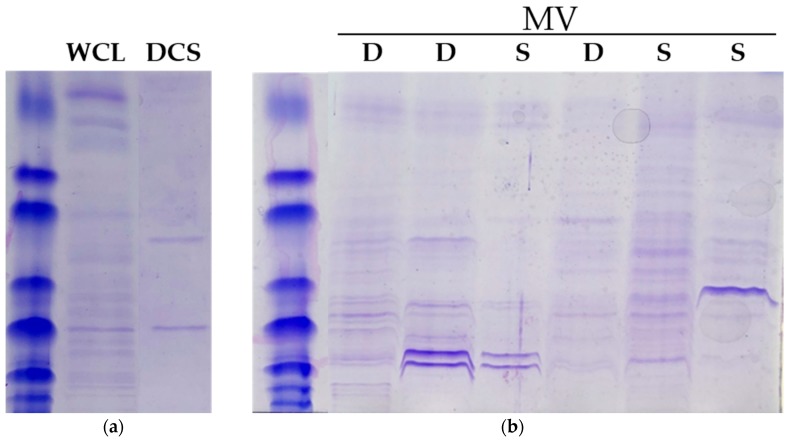
Protein content of ST45/USA600 *S. aureus* extracts. (**a**) Protein content of whole cell lysate and direct cell supernatant. Preparations from different isolates were virtually identical to each other. (**b**) Protein content of processed supernatants. Displayed are results from three independent strains isolated from decedents (D) and three independent strains isolated from survivors (S).

Strains recovered from decedents were indistinguishable from strains recovered from survivors in terms of growth rate (50 ± 4.2 min vs. 52 ± 5.7 min, *p* > 0.05), maximum stationary phase OD_600_ (12 ± 0.9 vs. 12 ± 1.4, *p* > 0.05) or colony pigmentation. Initially, two different methods for MV isolation were assessed. Processed liquid cultures required ultracentrifugation and multiple purification steps. Direct detection in cell culture supernatants passed through a submicron filter would represent a significant process improvement. Fluorescent signal determined from ultracentrifuge-purified MV was proportional to that determined directly from the cell culture supernatant—making cell culture supernatant a reasonable surrogate for MV detection. Furthermore, the fluorescent signal from the cell culture supernatant was statistically identical pre-filtration and post-filtration, suggesting that the small number of viable cells remaining in suspension provided a negligible contribution to the measurement (data not shown). As anticipated, both preparations passed through a submicron filter (direct supernatants or ultracentrifuge-processed supernatants) and were sterile when placed on agar growth medium, whereas unfiltered supernatants retained microbial viability. There were no differences in the protein content of the cell culture lysate or direct cell culture supernatant between strains recovered from decedents and strains recovered from survivors. Differences in the processed supernatant (MV) were modest and did not correlate with mortality (Figure 1). We selected unfiltered cell culture supernatants for further analysis.

**Figure 2:**
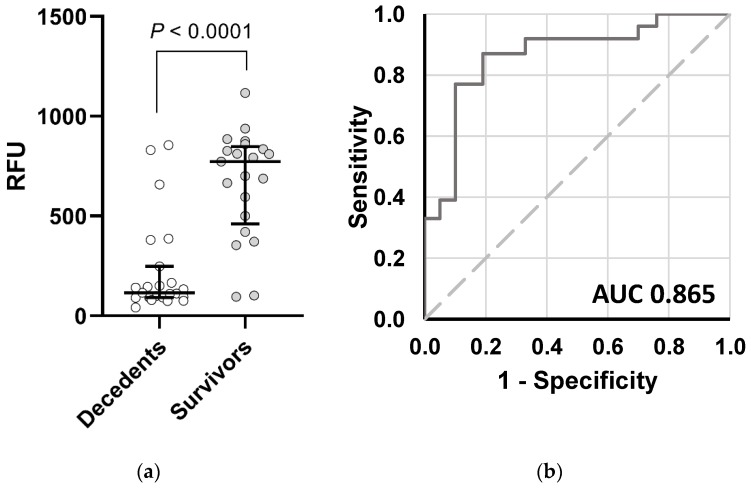
Membrane vesicle (MV) Production in ST45/USA600 *S. aureus*. (**a**) Clinical isolates from patients who die secondary to staphylococcal bacteremia (n = 23) produce fewer MV compared to patients who survive (n = 21, *p* < 0.0001). Data were quantified directly from cell culture supernatants. Outcomes are displayed as medians and interquartile ranges. RFU, relative fluorescence units. (**b**) Receiver Operating Characteristic (ROC) curve.

In contrast to other phenotypic characteristics, isolates from decedents produced significantly fewer MV (median 116 RFU, IQR 93-207) than isolates from survivors (median 773 RFU, IQR 501–836, *p* < 0.0001). A breakpoint of 301 RFU was identified by Classification and Regression Tree (CART) [12] as predictive of 30-day mortality (Figure 2). Using this breakpoint, the performance of MV to predict mortality exhibited a specificity of 90% (95% CI, 70–99%) and a sensitivity of 78% (95% CI, 56–93%). This breakpoint remained a significant predictor of mortality when controlling for age and source control (Table 1).

Although refinement of the sample preparation method has resulted in a more streamlined process, it still requires overnight growth in a liquid broth medium adding at least 24 h to sample assessment. We therefore explored whether differences in MV production could be identified directly from solid support media used in the clinical laboratory. Detection of MV directly from blood agar plates was not significantly different compared to MV recovered from stationary phase cultures (Wilcoxon signed rank, *p* = 0.98) and had perfect categorical agreement with overnight culture results.

## 3. Discussions

Endovascular staphylococcal infection is a complicated disease state, with a baseline mortality rate exceeding one in five patients [1]. Risk stratification for mortality has traditionally relied on patient factors (age, comorbid illnesses), process factors (source control time to appropriate antimicrobial therapy) and clinical diagnostics (immune biomarkers) [13]. However, the majority of these metrics overlook the impact of organism factors. Indeed, *S. aureus* contains an impressive diversity of metabolic potential, with over 80% of total staphylococcal genetic information present as an accessory genome, the components of which are found in only a subset of isolates [14]. The role of MV in coordinating effector delivery and mediating host–microbe interactions led us to hypothesize that they may be involved in the process of immune recognition of endovascular staphylococcal infection. As such, they could represent a patient-independent predictor of mortality. The present work establishes that, within the ST45/USA600 clade, low MV production is strongly associated with poor clinical outcome, presumably due to reduced microbe recognition and immune cell infiltration. This subdivision of MRSA is phylogenetically similar with minimal genetic variation between isolates. Furthermore, it is associated with a higher mortality rate compared to other sequence types and, as such, is more amenable to comparative analysis incorporating clinical outcome.

The deployment of MV in Gram-positive organisms appears to require, at a minimum, a membrane-deforming component such as a phenol-soluble modulin, and a cell wall degrading component such as staphylococcal lytic enzyme 1. Both systems are co-regulated by the accessory gene regulator system (agr) [11]. However, preliminary targeted genome sequence analysis does not identify any significant differences between isolates in the *agrABDC*, *sle1*, *sigAB* or *psmα1-4* sequences, suggesting that some other unknown factor is responsible for differences in MV production (data not shown). Therefore, the genetic differences between strains that result in differential MV production remain unclear and warrant additional, unbiased assessment.

The full isolation of MV represents a technical challenge, requiring multiple steps and specialty equipment that is not common in a routine clinical laboratory. It is also a multi-day procedure that limits its clinical utility as a tool to identify patients in need of more aggressive intervention. Here we present a straightforward, rapid and scalable technique to assess MV production that can be integrated into existing clinical microbiology workflows.

This study has several limitations. It establishes that clinical isolates differ in MV production but does not specifically link these differences to specific immune responses. Several in vivo models of staphylococcal infection exist, but animal models do not adequately capture human immune responses—a discrepancy thought to be the dominant factor in the clinical trial failure of a previous anti-staphylococcal vaccine candidate [15]. We also limited analysis to a specific clade of MRSA—ST45/USA600. We chose this in order to better control for variability in MV cargo between different *S. aureus* lineages, that may impact the immune recognition potency of MVs. Therefore, we anticipate the need for clade-specific breakpoint values. We do note, however, that the heterogeneity in protein content between ST45/USA600 MV preparations suggests that the amount of MV produced may be at least as important as the specific effectors present. Work is ongoing in our lab to characterize the host response to MV, and to determine the prognostic value of MV in predicting outcome with other *S. aureus* sequence types. This work supports the continued evaluation of MV production alongside patient factors in predicting the clinical outcome of endovascular *S. aureus* infection.

## 4. Materials and Methods

### 4.1. Study Design

For this study, we queried a retrospective biorepository maintained by one of our co-authors (MJZ), consisting of clinical patient information and the paired index *S. aureus* blood culture from sequential, non-duplicative adult inpatients admitted between 2005 and 2008 to Henry Ford Hospital—a tertiary, 800-bed hospital in Detroit, MI. In order to minimize the potential for confounding phylogenetic differences, we focused on ST45/USA600 MRSA, a lineage found in Detroit, Michigan that is associated with increased all-cause mortality [16]. Forty-four well-characterized sequential ST45/USA600 isolates with documented clinical outcomes [17] were identified and selected for this study. Of these 44 isolates, 21 were isolated from patients who survived the infection, and 23 were isolated from decedents. Isolates were confirmed to be ST45 by multi-locus sequence typing. Pertinent clinical data including patient age, ICU admission, Charleston Comorbidity Index, SOFA score, Pitt bacteremia score, time to appropriate antibiotic therapy, duration of bacteremia, adequate source control, Acute Physiology and Chronic Health Evaluation II (APACHE-II) score and 30-day all-cause mortality were recorded.

### 4.2. Strain Characterization

Growth rate was determined spectrophotometrically in 30 min intervals for overnight bacterial growth, subcultured into Tryptic Soy Broth (TSB). Bacteria were incubated with shaking (180 rpm) at 37 °C for a minimum of 6 h. The period of maximum growth was considered as the exponential growth phase, and a minimum of three consecutive values were used to calculate a rate. Bacterial pigmentation and colony morphology were assessed visually on Mueller–Hinton Agar, Brain Heart Infusion Agar and Blood Agar plates following 24 h of incubation at 35 °C.

### 4.3. Membrane Vesicle Isolation

Three methods for MV isolation were assessed. (*i*) Processed Cell Culture Supernatants. Clinical isolates were cultured overnight in Mueller–Hinton Broth (37 °C, 180 rpm). Overnight cell culture supernatants were clarified via centrifugation (5000× *g*, 10 min), filtered through a 0.2 µm membrane and ultracentrifuged (150,000× *g*, 90 min) to recover MV as described previously [18]. (*ii*) Direct Cell Culture Supernatants. Overnight cultures were centrifuged (5000× *g*, 10 min), supernatant transferred to a new tube and centrifuged again (5000× *g*, 10 min). Clarified cell culture supernatant was used directly for MV quantification. (*iii*) Direct From Colonies. Bacterial colonies were recovered directly from a blood agar plate and approximately 5 mg transferred into microfuge tubes. Cells were resuspended in normal saline (25 µL/mg dry cell weight), vortexed for 10 s and centrifuged twice as above. Clarified saline supernatant was used directly for MV quantification.

### 4.4. Membrane Vesicle Detection

Purified MV suspensions (100 µL) were quantified spectrofluorometrically as described previously, with modifications [19]. MV were detected on a SpectraMax M5 instrument using a membrane-specific styryl dye (FM1-43, Life Technologies Corporation, 5 µg/mL, ex/em 485/560 nm) that provides a greater resolution than the original FM4-64 dye, due to both increased quantum yield and improved vesicular membrane specificity [20]. Data are reported as relative fluorescence units. Assays were performed by S. Dey, who was blinded to clinical outcome data.

### 4.5. Protein Content Quantification

The protein content of the cell lysate, direct cell culture supernatant and processed cell culture supernatant was determined using the methods of Gurung et al. [21]. Normalized samples (equivalent to 1000 FM1-43 RFU) were separated by 12% SDS PAGE alongside a Precision Plus Protein Standard (Bio-Rad) and visualized with Coomassie Brilliant Blue R0250 (Bio-Rad). A minimum of three independent strains from decedents and three independent strains from survivors were evaluated.

### 4.6. Statistical Analysis

Descriptive data was expressed as mean and standard deviation, median and interquartile range, or frequencies and percentage. Univariable analysis was performed using a Student’s *t*-test, Wilcoxon rank sum, or Fisher’s Exact test for continuous, ordinal, and categorical data, respectively. Classification and Regression Tree (CART) methodology [12] was applied to identify a relative fluorescent unit breakpoint value predictive of 30-day mortality. Multiple logistic regression was performed using mortality as the dependent variable. Variables were initially considered for inclusion into the model if there was a difference between MV cutoff groups and there was an association with mortality (*p* < 0.2).

## 5. Conclusions

Low production of MV is associated with an increased risk of mortality in staphylococcal bacteremia caused by ST45/USA600 MRSA. MV can be rapidly quantified directly from blood agar plates, allowing for facile integration into existing clinical microbiology workflows.

## Figures and Tables

**Table 1:** Multiple Logistic Regression demonstrating association between membrane vesicles (>301 RFU) and mortality.

Variable	Odds Ratio (95% CI)	*p* Value
MV > 301 RFU	0.20 (0.04–0.91)	0.037
Age (years)	1.04 (0.99–1.09)	0.100
Source Control	0.35 (0.05–2.71)	0.314

## References

[1] Kourtis A.P., Hatfield K., Baggs J. (2019). Vital signs: Epidemiology and recent trends in methicillin-resistant and in methicillin-susceptible *Staphylococcus aureus* bloodstream infections—United States. MMWR Morb. Mortal. Wkly. Rep..

[2] Geriak M., Haddad F., Rizvi K., Rose W., Kullar R., LaPlante K., Yu M., Vasina L., Ouellette K., Zervos M. (2019). Clinical data on daptomycin plus ceftaroline versus standard of care monotherapy in the treatment of methicillin-resistant *Staphylococcus aureus* bacteremia. Antimicrob. Agents Chemother..

[3] Krishack P.A., Louviere T.J., Decker T.S., Kuzel T.G., Greenberg J.A., Camacho D.F., Hrusch C.L., Sperling A.I., Verhoef P.A. (2019). Protection against *Staphylococcus aureus* bacteremia-induced mortality depends on ILC2s and eosinophils. JCI Insight.

[4] Rose W.E., Shukla S.K., Berti A.D., Hayney M.S., Henriquez K.M., Ranzoni A., Cooper M.A., Proctor R.A., Nizet V., Sakoulas G. (2017). Increased endovascular Staphylococcus aureus inoculum is the link between elevated serum Interleukin 10 concentrations and mortality in patients With bacteremia. Clin. Infect. Dis..

[5] Minejima E., Bensman J., She R.C., Mack W.J., Tuan Tran M., Ny P., Lou M., Yamaki J., Nieberg P., Ho J. (2016). A dysregulated balance of proinflammatory and anti-inflammatory host cytokine response early during therapy predicts persistence and mortality in *Staphylococcus aureus* bacteremia. Crit. Care Med..

[6] Goldmann O., Medina E. (2018). *Staphylococcus aureus* strategies to evade the host acquired immune response. Int. J. Med. Microbiol..

[7] Smeltzer M.S. (2016). *Staphylococcus aureus* Pathogenesis: The Importance of Reduced Cytotoxicity. Trends Microbiol..

[8] Monecke S., Coombs G., Shore A.C., Coleman D.C., Akpaka P., Borg M., Chow H., Ip M., Jatzwauk L., Jonas D. (2011). A field guide to pandemic, epidemic and sporadic clones of methicillin-resistant *Staphylococcus aureus*. PLoS ONE.

[9] Lee E.Y., Choi D.Y., Kim D.K., Kim J.W., Park J.O., Kim S., Kim S.H., Desiderio D.M., Kim Y.K., Kim K.P. (2009). Gram-positive bacteria produce membrane vesicles: Proteomics-based characterization of *Staphylococcus aureus*-derived membrane vesicles. Proteomics.

[10] Liu Y., Defourny K.A.Y., Smid E.J., Abee T. (2018). Gram-positive bacterial extracellular vesicles and their impact on health and disease. Front. Microbiol..

[11] Wang X., Thompson S.D., Weidenmaier C., Lee J.C. (2018). Release of *Staphylococcus aureus* extracellular vesicles and their application as a vaccine platform. Nat. Commun..

[12] Breiman L., Friedman J.H., Olshen R.A., Stone C.J. (1984). Classification and Regression Trees.

[13] Minejima E., Mai N., Bui N., Mert M., She R.C., Nieberg P., Spellberg B., Wong-Beringer A. (2019). Defining the breakpoint duration of *Staphylococcus aureus* bacteremia predictive of poor outcomes. Clin. Infect. Dis..

[14] Bosi E., Monk J.M., Aziz R.K., Fondi M., Nizet V., Palsson B.Ø. (2016). Comparative genome-scale modelling of *Staphylococcus aureus* strains identifies strain-specific metabolic capabilities linked to pathogenicity. Proc. Natl. Acad. Sci. USA.

[15] McNeely T.B., Shah N.A., Frideman A., Joshi A., Hartzel J.S., Keshari R.S., Lupu F., DiNubile M.J. (2014). Mortality among recipients of the Merck V710 *Staphylococcus aureus* vaccine after postoperative *S. aureus* infections: An analysis of possible contributing host factors. Hum. Vaccin. Immunother..

[16] Moore C.L., Osaki-Kiyan P., Perri M., Donabedian S., Haque N.Z., Chen A., Zervos M.J. (2010). USA600 (ST45) methicillin-resistant *Staphylococcus aureus* bloodstream infections in urban Detroit. J. Clin. Microbiol..

[17] Sakoulas G., Guram K., Reyes K., Nizet V., Zervos M. (2014). Human cathelicidin LL-37 resistance and increased daptomycin MIC in methicillin-resistant *Staphylococcus aureus* strain USA600 (ST45) are associated with increased mortality in a hospital setting. J. Clin. Microbiol..

[18] Kim M.R., Hong S.W., Choi E.B., Lee W.H., Kim Y.S., Jeon S.G., Jang M.H., Gho Y.S., Kim Y.K. (2012). *Staphylococcus aureus*-derived extracellular vesicles induce neutrophilic pulmonary inflammation via both Th1 and Th17 cell responses. Allergy.

[19] Pader V., Hakim S., Painter K.L., Wigneshweraraj S., Clarke T.B., Edwards A.M. (2016). *Staphylococcus aureus* inactivates daptomycin by releasing membrane phospholipids. Nat. Microbiol..

[20] Stenovec M., Solajer R., Perdih A., Vardjan N., Kreft M., Zorec R. (2007). Distinct labelling of fusion events in rat lactotrophs by FM 1-43 and FM 4-64 is associated with conformational differences. Acta Physiol..

[21] Gurung M., Moon D.C., Choi C.W., Lee J.H., Bae Y.C., Kim J., Lee Y.C., Seol S.Y., Cho D.T., Kim S.I. (2011). *Staphylococcus aureus* produces membrane-derived vesicles that induce host cell death. PLoS ONE.

